# Neurostatus-SMARTCARE clinical trial: Enabling health care professionals to assess EDSS for decentralized trials in multiple sclerosis

**DOI:** 10.1177/13524585241305966

**Published:** 2024-12-20

**Authors:** Giulia Mallucci, Andrea Zimmer, Nikolaos Sfikas, Nuria Cerdá-Fuertes, Simon Wunderlin, Ioanna Athanasopoulou, Magdalena Mroczek, Vanny Phavanh, Lisa Sanak, Jolanda Suter, Bernadette Friedli, Jakob Kel, Thomas Trouillet, Sarah Simmen, Alex Ocampo, Wenjia Wei, Bernd Kieseier, Christian P Kamm, Ludwig Kappos, Marcus D’Souza

**Affiliations:** Research Center for Clinical Neuroimmunology and Neuroscience Basel (RC2NB), Basel, Switzerland; Neurocenter of Southern Switzerland, Regional Hospital of Lugano, Ente Ospedaliero Cantonale (EOC), Lugano, Switzerland; Research Center for Clinical Neuroimmunology and Neuroscience Basel (RC2NB), Basel, Switzerland; Novartis Pharma AG, Basel, Switzerland; Research Center for Clinical Neuroimmunology and Neuroscience Basel (RC2NB), Basel, Switzerland; Research Center for Clinical Neuroimmunology and Neuroscience Basel (RC2NB), Basel, Switzerland; Research Center for Clinical Neuroimmunology and Neuroscience Basel (RC2NB), Basel, Switzerland; Research Center for Clinical Neuroimmunology and Neuroscience Basel (RC2NB), Basel, Switzerland; Research Center for Clinical Neuroimmunology and Neuroscience Basel (RC2NB), Basel, Switzerland; Neurocenter, Lucerne Cantonal Hospital, Lucerne, Switzerland; Neurocenter, Lucerne Cantonal Hospital, Lucerne, Switzerland; Neurocenter, Lucerne Cantonal Hospital, Lucerne, Switzerland; Research Center for Clinical Neuroimmunology and Neuroscience Basel (RC2NB), Basel, Switzerland; Research Center for Clinical Neuroimmunology and Neuroscience Basel (RC2NB), Basel, Switzerland; Research Center for Clinical Neuroimmunology and Neuroscience Basel (RC2NB), Basel, Switzerland; Novartis Pharma AG, Basel, Switzerland; Novartis Pharma AG, Basel, Switzerland; Novartis Pharma AG, Basel, Switzerland; Neurocenter, Lucerne Cantonal Hospital, Lucerne, Switzerland; Department of Neurology, Inselspital, Bern University Hospital and University of Bern, Bern, Switzerland; Research Center for Clinical Neuroimmunology and Neuroscience Basel (RC2NB), Basel, Switzerland; Research Center for Clinical Neuroimmunology and Neuroscience Basel (RC2NB), Basel, Switzerland; Department of Neurology, University Hospital Basel, Basel, Switzerland

**Keywords:** Multiple sclerosis, EDSS, decentralized trials, health care professionals, clinical trials

## Abstract

**Background:**

Neurostatus-Expanded Disability Status Scale (EDSS) is the standard measure used to assess impairment and disability in multiple sclerosis (MS) trials but requires trained expert neurologists.

**Objectives:**

This study aims to evaluate the concordance of Neurostatus-EDSS assessments from specially trained health care professionals (HCPs) and standardized trained neurologists.

**Methods:**

A Swiss multicenter, randomized, cross-over study with 100 people with MS. HCPs were trained to assess the Neurostatus-EDSS based on the newly developed SMARTCARE-EDSS training method.

**Results:**

The concordance rate between HCPs and neurologists was 0.87 (95% confidence interval (CI) = 0.815–0.925).

**Conclusion:**

Trained HCPs can reliably perform Neurostatus-EDSS assessments, supporting broader implementation and improved trial access.

## Introduction

The monitoring of people with multiple sclerosis (pwMS) in clinical routine requires standardized grading of symptoms over time. The primary tool in clinical phase II/III trials and registries such as the Swiss Multiple Sclerosis Cohort (SMSC) and MSBase is the Neurostatus-EDSS.^1^ In 2009, a digital version providing algorithm-based consistency checks, the Neurostatus-eEDSS, was introduced.^2^ The shift toward decentralized clinical trials (DCTs) necessitates accurate remote assessment tools.^3^ DCTs can broaden participant inclusion, reduced patient burden, and increased efficiency. Reliable remote assessments must capture clinical symptoms with the consistency of in-clinic evaluations. However, remote neurological examinations are challenging. Neurostatus-EDSS requires detailed physical exams and interviews, demanding high expertise, which is difficult to replicate remotely. To provide these aspects, we developed the smartly modernized assessment-recorded, telemedical, care professional-assisted, remotely evaluated Neurostatus-SMARTCARE, designed for the DCT environment. It enables specially trained health care professionals (HCPs) to perform the Neurostatus-EDSS equal to neurologists. In this study, HCPs received the newly developed SMARTCARE-training to assess the Neurostatus-EDSS. The primary aim of the study was to evaluate the concordance between Neurostatus-EDSS assessments performed by SMARTCARE-trained health care professionals and those conducted by neurologists trained in a standardized manner.

## Methods

### Study design

**Figure 1.**
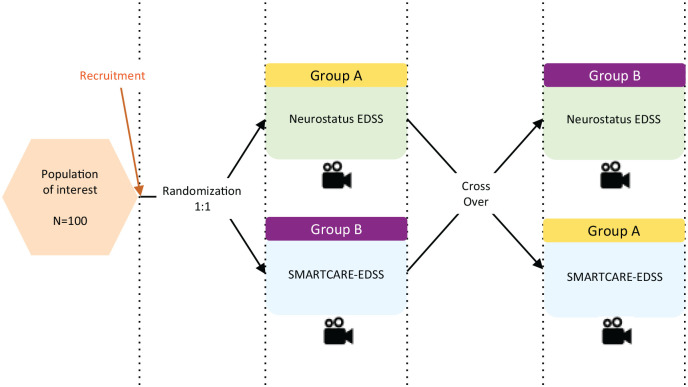
Study design. All assessments were video recorded for quality assurance and independent review.

A randomized, cross-over study was conducted at two Swiss tertiary care sites: University Hospital Basel and Cantonal Hospital Lucern. The pwMS were randomized 1:1 to group A or group B. Group A was first assessed by neurologists who received a standard Neurostatus-EDSS training, followed by HCPs who received the SMARTCARE-training, group B vice versa (Figure 1 shows study design). The training schedule is available in Supplementary Material. All assessments were performed at the respective hospitals. We employed a randomized cross-over design for this study to minimize variability and reduce potential biases related to differences in patient characteristics and order of assessments. This design allowed each participant to serve as their own control, by being assessed by both a neurologist and an SMARTCARE-trained HCP. This design also controlled for any potential order effects, as assessments were conducted in both sequences (neurologist first or HCP first), ensuring a balanced and unbiased evaluation of concordance. After a full neurological examination, the Neurostatus subscores, Functional System Scores (FSS), the Ambulation Score (AS), and the EDSS step were determined according to the Neurostatus-EDSS definitions via a Neurostatus-eEDSS Web-Interface.^4^ No real-time feedback via the Neurostatus-eEDSS Web-interface was provided to allow an unbiased comparison between HCPs and neurologists. The HCPs and neurologists were blinded to each other’s findings and assessment scores.

### Study population

The study was conducted between December 2022 and March 2024. Participating four HCPs and nine neurologists were certified at level C in Neurostatus-EDSS prior to conducting any neurological evaluations with patients. The HCPs received their certification after completing the SMARTCARE-training, whereas the neurologists had already been certified before the study began.

The pwMS had a confirmed MS diagnosis according to the revised McDonald 2017 criteria,^5^ and at least one EDSS assessment within 2 years before enrolment. Exclusion criteria included limited language proficiency, specifically in German, as this was the primary language used for patient interactions and assessments in the study. Stratified sampling based on EDSS ensured a representative sample across different disability grades.

### Statistical analysis

The primary endpoint was the concordance rate between the neurologist’s Neurostatus-EDSS assessment and the HCP’s Neurostatus-EDSS assessment. Ratings of neurologists and HCPs were defined as concordant if either (1) the EDSS steps were identical or (2) the EDSS steps laid between values as shown in the Supplementary Table 1.

We hypothesized that the concordance rate was significantly higher than an unacceptance level of 80%, under a one-sided test with a type-I error rate (α) of 0.05. We calculated the concordance rate and its 95% confidence interval (CI) based on the normal approximation (Wald confidence interval). In addition, two alternative more conservative methods to calculate 95% CI were used as sensitivity analyses—Wilson confidence interval and Clopper–Pearson confidence interval.

### Ethical considerations

The study was approved by the ethics committee of Northwestern and Central Switzerland (ID: 2022-01294), conducted in accordance with ethical standards and registered at ClinicalTrials.gov: NCT05575843.

## Results

Totally, 102 pwMS were enrolled, and two dropped out before the visit; therefore, finally, 100 were included in the analyses (study population) with available EDSS assessments conducted by both neurologists and HCPs. Patients’ characteristics are provided in Table 1.

**Table 1.** Demographical and clinical characteristics of the study population.

Characteristics	Patients (*N* = 100)
Age (years) (mean ± SD)	51.31 ± 13.58
Sex, *n* (%)	
Male	44 (44.0)
Female	56 (56.0)
Baseline EDSS (mean ± SD)/ *n* (%)	3.36 ± 2.01
0-2.5	38 (38.0)
3-5.5	41 (41.0)
>5.5	21 (21.0)
Duration of MS since diagnosis (years) (mean ± SD)	12.94 ± 10.37
Duration of MS since disease onset (years) (mean ± SD)	16.68 ± 12.28
MS subtype, *n* (%)	
RRMS	81 (81.0)
SPMS	9 (9.0)
PPMS	8 (8.0)
Other	2 (2.0)
Site, *n* (%)	
Basel	79 (79.0)
Luzern	21 (21.0)
Randomization group at visit 1, *n* (%)	
A: first neurologist, second HCP	49 (49.0)
B: first HCP, second neurologist	51 (51.0)

EDSS: Expanded Disability Status Scale; HCP: health care professional; MS: multiple sclerosis; RRMS: relapsing remitting multiple sclerosis; PPMS: primary progressive multiple sclerosis; SD: standard deviation; SPMS: secondary progressive multiple sclerosis.

The concordance rate of EDSS steps between HCP and neurologist is 0.87 (95% CI = 0.815–0.925). For sensitivity analyses: (a) Wilson confidence interval = (0.805–0.916) and (b) Clopper–Pearson confidence interval = (0.801–0.921). We can conclude that the concordance rate of EDSS steps between HCP and neurologist is higher than 80%.

In more detail, across different raters, the variability was fairly consistent. Omitting one rater with only two assessments performed, the standard deviations of the 9 raters remaining had an interquartile range between 1.80 and 1.96. This means half the rater’s ratings had average variabilities spanning only 0.16 points on EDSS scores from 0 to 8.5, suggesting relatively similar inter-rater variability.

## Discussion

In this study, conducted in two Swiss MS centers, we evaluated the concordance of Neurostatus-EDSS assessments performed by neurologists and HCPs receiving a new developed SMARTCARE-training. The concordance rate of our study demonstrates that HCPs can perform a standardized neurological examination and determine the Neurostatus-EDSS equal to neurologists. As the “SMARTCARE-EDSS” assessment is designed to be performed by specially trained HCPs at patients’ home, this allows for a more decentralized assessment.

These results of the first analysis from the SMARTCARE study will have a crucial impact on future study protocols of phase II/III trials for pwMS or related disorders. The option of reliably and accurately assessing patients’ condition in their own environment would allow for a broader population to participate in clinical trials and—where appropriate—more frequent assessments. While telephone-based EDSS assessment has been previously validated^6^ and offers certain logistical advantages, the SMARTCARE-EDSS provides a more comprehensive assessment by incorporating a full physical examination alongside the patient interview. This approach is especially critical in detecting subtle clinical changes that may be missed in a purely interview-based assessment. However, we recognize that the EDSS, while widely used, has limitations in its sensitivity and ability to capture all aspects of disability, particularly in more disabled pwMS. As modern MS trials increasingly combine EDSS with other clinical measures, such as the Symbol Digit Modalities Test (SDMT), 9-Hole Peg Test (9HPT), and Timed 25-Foot Walk Test (T25FWT), future efforts will need to focus on integrating more sensitive and multidimensional tools, specifically digital measures^7,8^ to improve the assessment of patient outcomes.

In future trials, such decentralized high-quality neurological assessments would complement and anchor patient-reported outcomes^9^ as well as digital applications, currently in development^8,10,11^ and limiting the time spent for visiting MS centers. Furthermore, when patients are unable to come to the respective MS centers for routine checks (e.g. due to severe mobility restrictions or a pandemic situation like COVID-19), the “SMARTCARE-EDSS” can provide a sufficient clinical evaluation of the patient’s status remotely in clinical routine. Additional analyses of our study data will also assess whether discordance between raters is limited to specific subscores or FSS of EDSS, or if this variation occurs across the spectrum of the evaluations.

A limitation of this study was the participation of only two MS centers and the focus on nurses as HCPs. Future research should explore the involvement of other HCPs, such as physiotherapists. To provide for the best possible and unbiased comparison, all assessments were performed in the participating MS centers and not at patients’ homes. As no specific equipment unique to the MS clinics was required, the results should nevertheless be transferable to home visits.

## Supplemental Material

sj-docx-1-msj-10.1177_13524585241305966 – Supplemental material for Neurostatus-SMARTCARE clinical trial: Enabling health care professionals to assess EDSS for decentralized trials in multiple sclerosis Supplemental material, sj-docx-1-msj-10.1177_13524585241305966 for Neurostatus-SMARTCARE clinical trial: Enabling health care professionals to assess EDSS for decentralized trials in multiple sclerosis by Giulia Mallucci, Andrea Zimmer, Nikolaos Sfikas, Nuria Cerdá-Fuertes, Simon Wunderlin, Ioanna Athanasopoulou, Magdalena Mroczek, Vanny Phavanh, Lisa Sanak, Jolanda Suter, Bernadette Friedli, Jakob Kel, Thomas Trouillet, Sarah Simmen, Alex Ocampo, Wenjia Wei, Bernd Kieseier, Christian P Kamm, Ludwig Kappos and Marcus D’Souza in Multiple Sclerosis Journal

sj-docx-2-msj-10.1177_13524585241305966 – Supplemental material for Neurostatus-SMARTCARE clinical trial: Enabling health care professionals to assess EDSS for decentralized trials in multiple sclerosis Supplemental material, sj-docx-2-msj-10.1177_13524585241305966 for Neurostatus-SMARTCARE clinical trial: Enabling health care professionals to assess EDSS for decentralized trials in multiple sclerosis by Giulia Mallucci, Andrea Zimmer, Nikolaos Sfikas, Nuria Cerdá-Fuertes, Simon Wunderlin, Ioanna Athanasopoulou, Magdalena Mroczek, Vanny Phavanh, Lisa Sanak, Jolanda Suter, Bernadette Friedli, Jakob Kel, Thomas Trouillet, Sarah Simmen, Alex Ocampo, Wenjia Wei, Bernd Kieseier, Christian P Kamm, Ludwig Kappos and Marcus D’Souza in Multiple Sclerosis Journal
