# Supplementary material for: Neurostatus-SMARTCARE clinical trial: Enabling health care professionals to assess EDSS for decentralized trials in multiple sclerosis
Source: Mult Scler. 2024 Dec 20;31(4):497–501. doi: 10.1177/13524585241305966 (PMC11956382; doi:10.1177/13524585241305966)
Supplement: sj-docx-1-msj-10.1177_13524585241305966 – Supplemental material for Neurostatus-SMARTCARE clinical trial: Enabling health care professionals to assess EDSS for decentralized trials in multiple sclerosis [file sj-docx-1-msj-10.1177_13524585241305966.docx]

**EDSS Training SMARTCARE Study**

**Neurologists**

Receive a standard training:

- 60 - 90 minutes of individual, virtual training with an Neurostatus-EDSS expert according to the standards of trainings provided in phase 2 and 3 clinical MS trials.
- Individual work through the Neurostatus-DVD
- EDSS Certificate Level C
- Introduction in Data entry into the Neurostatus web interface, onsite or virtual

**Non-neurologist HCPs**

Nurses receive a specialized (SMARTCARE) training with an Neurostatus-EDSS expert:

***1. 3-Day Training, over 2 weeks, each day approximately 7 hours (totally 21 hours):***

- Anatomy/Physiology related to the body functions assessed in the EDSS
- General neurological examination
- Examination techniques/methodology for the EDSS
- Scoring of findings
- Sequence of examination
- On the third day: Each learner must conduct one complete examination as a practice

***2. Self-Study, over 3 weeks (8 hours):***

- Examine 5 – 10 healthy individuals
- Independently review examination techniques and scoring

***3. Training Session (2 hours, onsite or virtual):***

- 1 hour: Questions about examination/scoring from the self-study
- 1 hour: Entering EDSS parameters into the Neurostatus web interface

***4. Practical Exercise, over 3 weeks (8 hours):***

- Work through Neurostatus-DVD (4 hours)
- Practice and score on 3 - 5 patients in clinical practice under the guidance of a treating neurologist (6 hours)

***5. Finalization, over 2 weeks (6 hours):***

- Training session with questions about patient examinations (2 hours)
- EDSS Certificate Level C (2 hours)
- 1 EDSS on a healthy person with video recording, review and feedback by expert (2 hours)

**Total: 45 hours over 10 weeks**

**6. Re-Training**

- Re-training session (onsite or virtual) after 3 months and after 6 months (2 hours each)
- Questions and answers on patient examinations practiced
