# Supplementary material for: Neurostatus-SMARTCARE clinical trial: Enabling health care professionals to assess EDSS for decentralized trials in multiple sclerosis
Source: Mult Scler. 2024 Dec 20;31(4):497–501. doi: 10.1177/13524585241305966 (PMC11956382; doi:10.1177/13524585241305966)
Supplement: sj-docx-2-msj-10.1177_13524585241305966 – Supplemental material for Neurostatus-SMARTCARE clinical trial: Enabling health care professionals to assess EDSS for decentralized trials in multiple sclerosis [file sj-docx-2-msj-10.1177_13524585241305966.docx]

| **EDSS-step** | **EDSS-step** |  | **Assessed as** |
| --- | --- | --- | --- |
| 0 | 1.0 |  | concordant |
| 0 | 1.5 | and above | different |
| 1.0 | 1.5 |  | concordant |
| 1.0 | 2.0 | and above | different |
| 1.5 | 2.0 |  | concordant |
| 1.5 | 2.5 | and above | different |
| 2.0 | 2.5 |  | concordant |
| 2.0 | 3.0 | and above | different |
| 2.5 | 3.0 |  | concordant |
| 2.5 | 3.5 | and above | different |
| 3.0 | 3.5 |  | concordant |
| 3.0 | 4.0 | and above | different |
| 3.5 | 4.0 |  | concordant |
| 3.5 | 4.5 | and above | different |
| 4.0 | 4.5 |  | concordant |
| 4.0 | 5.0 | and above | different |
| 4.5 | 5.0 |  | concordant |
| 4.5 | 5.5 | and above | different |
| 5.0 | 5.5 |  | concordant |
| 5.0 | 6.0 | and above | different |
| 5.5 | 6.0 | and above | different |
| 6.0 | 6.5 | and above | different |
| 7.0 | 7.5 | and above | different |
| 7.5 | 8.0 | and above | different |

*Table 1 supplementary: Concordance table depending on EDSS step. Abbreviations: EDSS expanded disability status scale.*
